# Electroosmotic flow in soft clay and measures to promote movement under direct current electric field

**DOI:** 10.1371/journal.pone.0302150

**Published:** 2024-04-16

**Authors:** Zhaohua Sun, Cheng Zhang, Beukes Demarscho Eugene, Xiwen He

**Affiliations:** 1 School of Transportation and Civil Engineering, Nantong University, Nantong, China; 2 Key Laboratory of New Technology for Construction of Cities in Mountain Area, Ministry of Education, Chongqing University, Chongqing, China; The British University in Egypt, EGYPT

## Abstract

Electroosmosis has been proposed as a technique to reduce moisture and thus increase the stability of soft clay. However, its high energy consumption and uneven reinforcement effect has limited its popularization and application in practical engineering. This paper presents the results of some electrokinetic tests performed on clayey specimens with different electrification time and anode boundary conditions. The results indicate that the timing of the formation of electroosmotic flow (EF) by the water originally contained in different soil cross sections, from the anode to the cathode, varies. The measuring soil cross section nearest the anode first reached the limiting water content of 22%±3% and electroosmosis had to be stopped. Water injection into the anode during electroosmosis enhanced further drainage of other four measuring soil cross sections until the second soil cross section from the anode reached the limiting water content of 30%±2%. Electroosmosis with water injection into the anode technique provides more uniform reinforcement, increasing EF, and environmental protection. The experimental results highlighted the relevant and expected contribution of water injection into the anode on the effectiveness of the electroosmotic treatment as a soft clay improvement technique.

## Introduction

There are designing and constructing problems in civil engineering structures, like road embankments, over soft soils as a result of the high compressibility, high water content, and low shear strength of the soils [1]. Electroosmosis has been considered to be a very promising drainage technology for soft clay foundation treatment [2]. It was first practically applied in the stabilisation of railway excavation slope by Casagrande [3]. Following this successful field application, electroosmosis has become an established technique for foundation reinforcement, dam stalilization, environmental geotechnical engineering [4]. A series of complex physical and chemical reactions, such as electroosmotic flow (EF), electrophoresis, ion exchange and migration, electrode redox reaction, and cementation, take place in the soft clay under direct current field [5, 6]. The reinforcement mechanism of electroosmosis for soft clay with high water content, low permeability, fine particles, and large specific surface area can be summarized mainly as dewatering consolidation and chemical strengthening. Water drainage achieved by the movement of EF in the soil from the anode to the cathode is the dominant electroosmosis process accountable for the variation in soil strength [7, 8].

In low permeability soils, the EF is much more efficient than hydraulic flow. The EF depends on fluid characteristics (dielectric constant and viscosity) and soil surface characteristic represented by zeta potential, as well as voltage gradient [9]. In other words, the velocity of EF is mainly related to the coefficient of electro-permeability of the soil and the gradient of applied electric potential [10–12]. The coefficient of electro-permeability is not sensitive to soil particle size, and smaller electric potential gradients can produce larger pore water seepage. Hence, soft clay can be quickly drained and consolidated by electroosmosis method [13]. However, the soil reinforcement effect is considerably uneven due to the unidirectional EF from the anode to the cathode. Significant reduction of soil water content near the anode increases the interfacial resistance between soil and electrodes, which reduces the energy consumption efficiency of electroosmosis. The high energy consumption and uneven reinforcement effects have become the main technical bottlenecks of electroosmosis. It is crucial to improve the reinforcement quality and efficiency of electroosmosis and reduce energy consumption and cost through technological innovation and mechanism research, which is of great significance to promote the engineering application of electroosmosis. Considering measures to promote EF is an effective way to improve the reinforcement effect of electroosmosis method.

There are many ways to promote EF, such as suitable voltage gradients [14], stepwise movement of anode [15], and incorporating with vacuum preloading or other methods [16]. However, the latter two methods require high labour intensity in practical engineering applications. Importantly, the material of the electrode such as metal, graphite, carbon and reusable electrokinetic geosynthetics has a significant impact on the effect of electroosmosis [17]. Moreover, the EF can be enhanced by the addition of chemical agents. The intensity of EF depends on the character, concentration, and pH of the chemical solution, as well as the character of soil and the voltage applied [18]. For example, the amount of drained water from the cathode for electroosmosis with injection of saline solutions was about 1.4 times that for electroosmosis only [19]. The increase of cation in the soil due to the injection of chemical solutions will result in an increase of electric conductivity and hydration of cation, which causes more absorbed water to migrate towards the cathode [20]. EF plays an important role in contaminated soil, because the development and maintenance of a high EF is beneficial for the removal of contaminants from soils. Popov et al. [21] demonstrated that chelating agents are capable of providing an one order of magnitude increase in electroosmotic flow intensity. The use of citric acid induced large EF due to the interaction of the organic acids with the soil particles [9]. Unfortunately, the addition of chemical agents may cause environmental problems since they are used in large quantities. Hence the need to develop an eco-friendly and economical enhancement method. Continuous injection of water into the anode may after all be accepted as a good choice for EF enhancement [7, 8].

EF varies with time during electrokinetic processes. Numerous determinations were made of the soil real-time water content at different distances from the electrodes, water discharge at various intervals of time, and strength after the treatment in order to study the dewatering process and the soil strengthening effect of electroosmotic treatment [3, 7, 8, 22]. However, there are still mechanisms that remain to be explained such as the transport trajectory of EF in different soil segments and time stages, and its effect on the increasing process of shear strength.

In order to analyse the transport trajectory of EF in the soil and provide more uniform reinforcement, energy conservation, water discharge increase, and environmental protection operating conditions for the electroosmotic treatment of soft clay, a series of laboratory experiments with different electrification time and anode boundary conditions have been performed. The main goal of the paper is to highlight the role of EF and water injection into the anode during electroosmosis. The planned experimental activity consists of a series of tests to for the water discharge, electric potential, water content, and shear strength. These parameters have been monitored during different stages of the EO experiment to get an idea of the extent of changes. Finally, to verify the uniformity of electroosmosis with water injection into the anode treatment as soft clay improvement technique, the Atterberg limits and the coefficient of variation for water content and vane shear strength were evaluated.

## Materials and test schemes

### Materials

The experimental model box was made of transparent polymethyl methacrylate with internal dimensions of 300 mm×100 mm×100 mm. Electric vertical drains (EVDs) were chosen as the electrodes, which consisted of pipe body, copper wire, and filter cloth. The pipe body is made of polyethylene, carbon black, and graphite with good conductivity and corrosion resistance [23]. Two strands of copper wire buried in the pipe body along the vertical direction. Several drainage grooves were distributed along the vertical direction of the outer wall of the pipe body, and drainage holes were evenly perforated besides the drainage grooves all around the pipe body. The filter cloth was wrapped around the pipe body. The inner and outer diameter of tubular EVD is 17 mm and 27 mm, respectively. Direct-current power supply (RXN-605D) has a digital display feature and steady output voltage with maximum output power of 60 V×5 A. Rubber-insulated copper electric wires were used to connect the electrodes and DC power supply.

A drainage system was used to pump out the water that collected at the cathode. One end of the transparent polyurethane tubing with outer diameter of 8 mm was inserted into the bottom of the cathode tube, and another end was connected to a 1000 mL collecting bottle with minimum scale of 1 mL. The collecting bottle was connected with a water circulating multi-purpose vacuum pump (SHB-IIIA). The power, maximum vacuum degree, and single tap air pumping amount of the vacuum pump is 180 W, 0.085 MPa, and 10 L/min, respectively.

The clay powder used for these tests was purchased from of a mining company in Nanjing, China. Geotechnical tests were performed basing on canonical standard ASTM, as shown in Table 1. The plasticity index of the soil was 22. Sieving and hydrometer methods were carried out to analyse the soil particle size. The percentage of particle grain size less than 0.075 mm and 0.005 mm was 85.2% and 36.0%, respectively. According to ASTM D2487-06 classfication, the soil samples are classified as clay of low plasticity symbolised by CL.

**Table 1:** Properties of clay powder used in the experiments.

Soil	Specific gravity	Permeability coefficient (cm/s)	Liquid limit (%)	Plastic limit (%)	Particle size analysis		
					Sand (%)	Silt (%)	Clay (%)
Clay soil	2.73	3.0×10^−7^	43	21	15	49	36

### Test schemes

**Fig 1:**
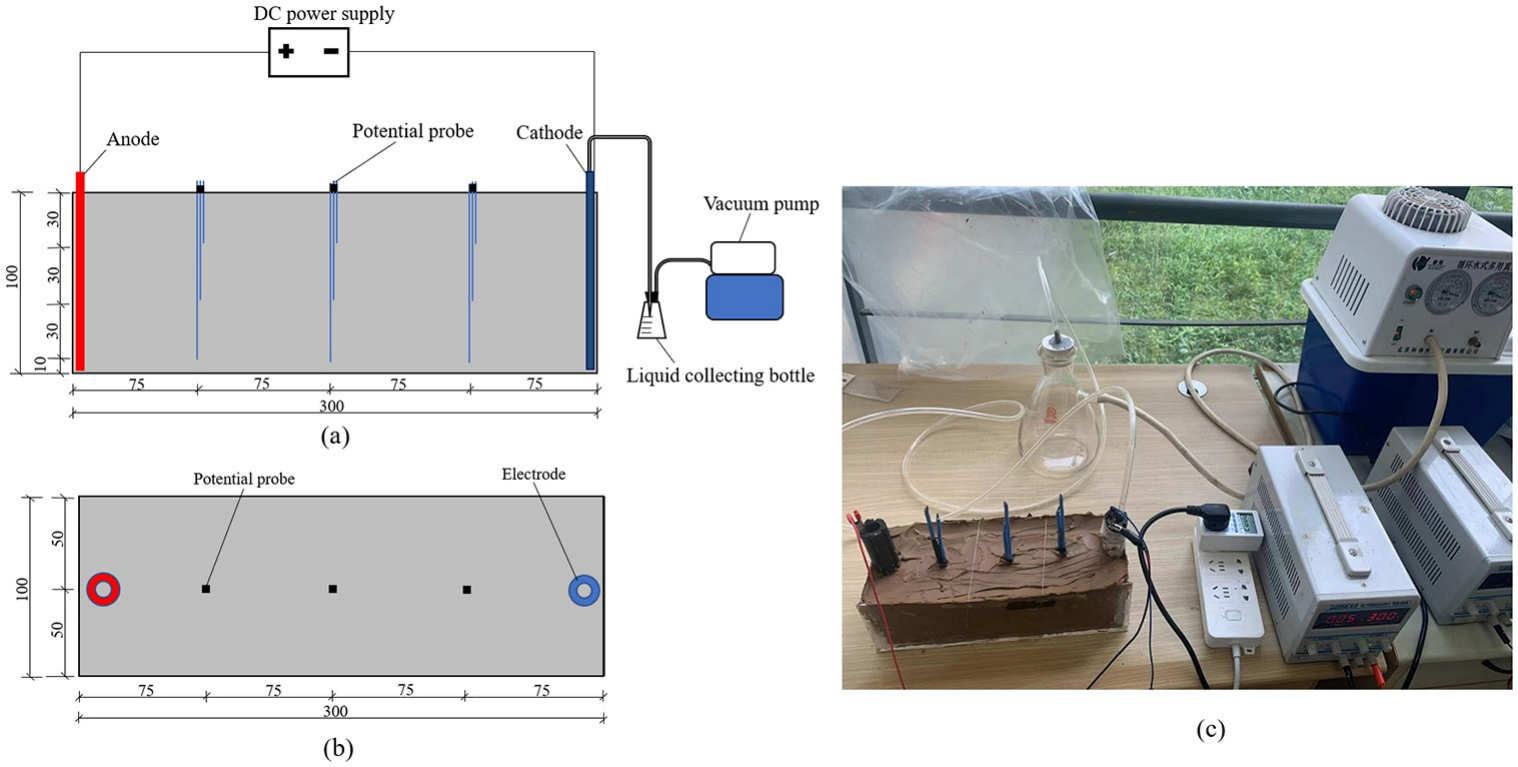
Experimental setup of electroosmosis. a) Side view. b) Plan view. c) Real image (unit mm).

There are many factors, such as electrode material, soil properties, electric potential gradient, electrode spacing etc., that influence the experimental results. These experiments are selected representatively through test conditions to carry out and analyse the test law. Although it cannot be generalized, it is still of certain reference significance as a typical representative. Table 2 shows the test programs and conditions. For each test 3.55 kg of clay soil powder with 8.7% initial water content was measured and mixed with 1.34 kg of distilled water by a mechanical mixer to reach 50% target water content. The actual water content of the soil for each test is shown in Table 2. The mass of soil samples for each test was 4.89 kg. The soil sample was slowly layered and gently pressed in the test device to get rid of air bubbles. The height of the soil sample in each test before treatment was about 100 mm. Two electrodes were inserted into the soil at both ends of the length direction and nine potential probes were inserted into the tripartite position of the soil sample with 30 mm, 60 mm, and 90 mm depth, as shown in Fig 1. In the existing studies, the electric potential gradient applied on soft clay is usually between 0.25 and 5 V/cm [24]. A typical voltage of 30 V was applied to each test and the electric potential gradient was about 1 V/cm.

**Table 2:** Test schemes.

Test number	Initial soil water content (%)	Total electrification time (h)	Water injection into the anode	Intermittent electrification mode	Soil mass (kg)	Applied voltage (V)
**T1**	50.5	1	-	-	4.89	30
**T2**	50.5	2	-	-		
**T3**	50.6	4	-	-		
**T4**	50.6	8	-	-		
**T5**	50.6	12	-	-		
**T6**	50.6	24	-	Intermittent 12 h after continuous power on 12 h per time		
**T7**	50.6	36	Injection 2 mL distilled water into the anode when the drainage rate lower than 2 mL/h each time			
**T8**	50.8	72	Injection 8 mL distilled water into the anode after each continuous power on 12 h each time			

Eight EO tests were carried out with different electrification time. The total electrification time of T1 was 1 h. That increased to 2 h, 4 h, 8 h, 12 h and 24 h for T2 to T6 without water injection into the anode (ordinary electroosmosis). Moreover, the electrification time of T6 was determined by its drainage rate. This means that when the soil experiences one more hour of electroosmosis and no water can be drained out from the cathode, it could be terminated. In order to further promote the water discharge of the soil for T7, when the drainage rate was lower than 2 mL/h, 2 mL distilled water was injected into the anode each time. For T8, 8 mL distilled water was injected into the anode after each 12 h of continuous power. For T6, T7, and T8, intermittent electrification mode, which was used to counterbalance pore water pressure difference, that was intermittent 12 h after continuous power of 12 h per time was used.

**Fig 2:**
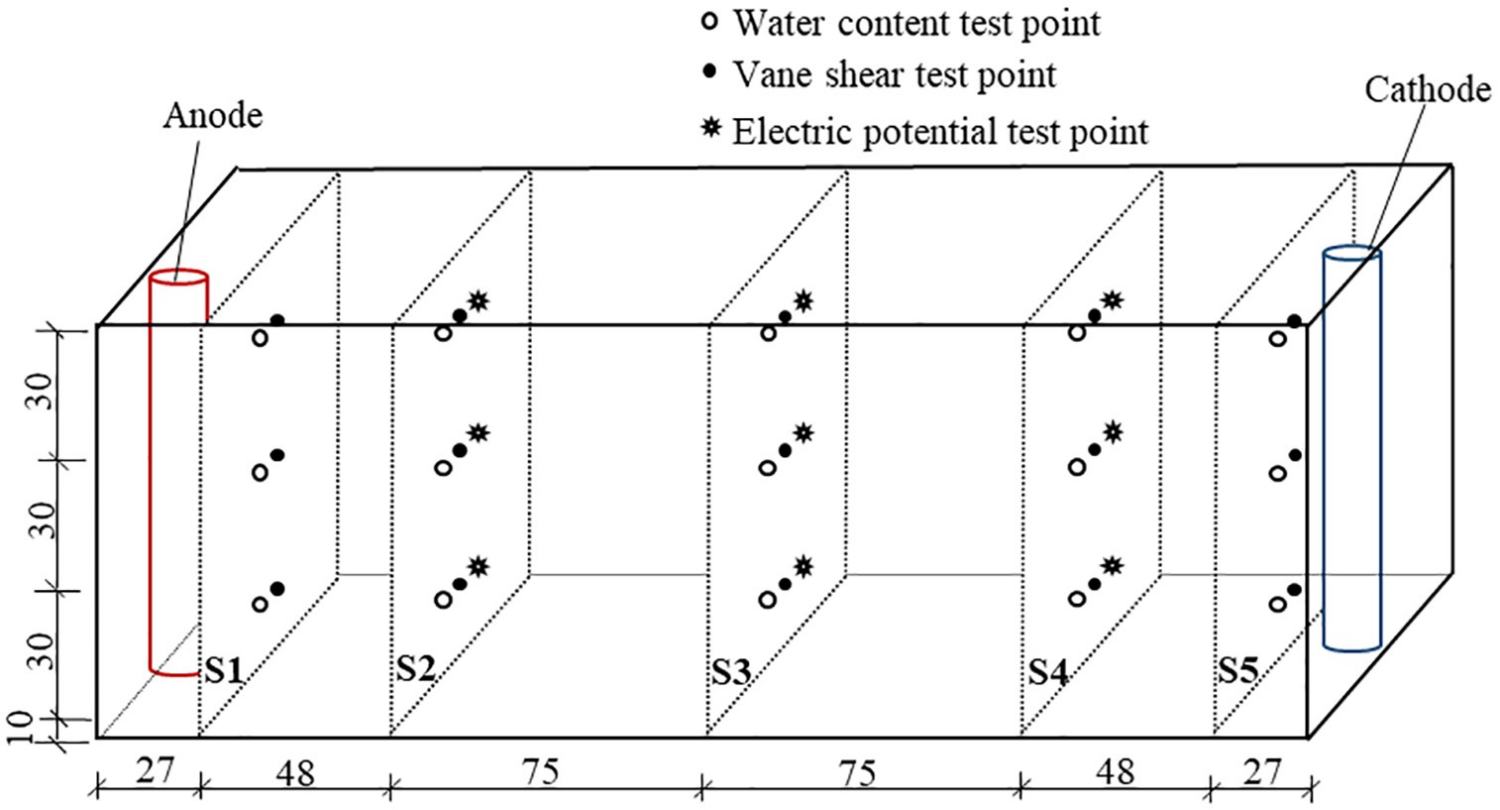
Cross sections represented as S1-S5 for voltage potential, vane shear test and water content (unit mm).

The water discharge, electric current and electric potential was monitored during the experiments. In order to facilitate the analysis of the change rule of monitoring datas, the interval time of T6-T8 is not reflected in their data result graphs. After treatment, the soil was divided into six segments, the water content and shear strength at soil cross sections of S1 to S5 was tested. The locations for monitoring and testing are shown in Fig 2. The strength tests confirming to ASTM 2001 were conducted by dynamoelectric vane shear (TT-LVS) manufactured by Zhejiang Geotechnical Instrument. The vane shear apparatus has blades which are 25.4 mm in diameter, 25.4 mm in height, and 0.01 mm in thickness. The values of the shear stress and rotation angle were automatically recorded on a computer. Moreover, the Atterberg limits of the soil after treatment near the anode, cathode, and the middle location between the electrodes (S3 section) were tested.

## Results

### Water discharge

**Fig 3:**
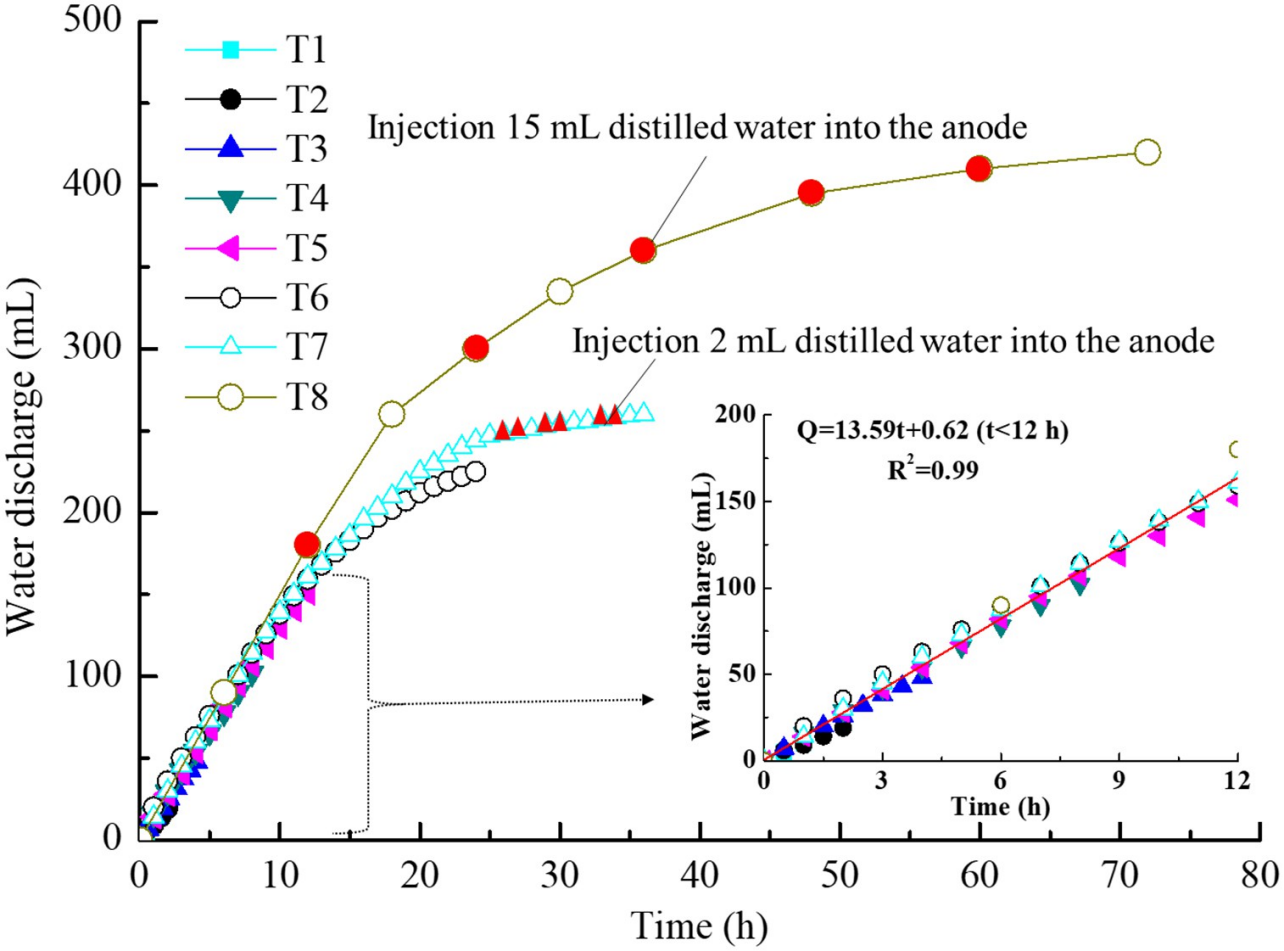
Water discharge for each test.

Fig 3 illustrates the changes in water discharge for each test. For T1 to T7, their water discharge curves along with time before 24 h almost has the same trend. At the end of T7 test injection of 2 mL distilled water into the anode for six times, only 23 mL more water than T6 was discharged in the soil. For T8, injection 8 mL distilled water into the anode after each continuous power of 12 h each time significantly improved the water discharge of the soil. After removing the volume of injected water a total of 380 mL of water, approximately 108 mL higher than that of T7, was drained out. Since the first injection at hour 12, the drainage curve of T8 was higher than that of other tests. The water discharge curves of each test demonstrated that the results have a high repeatability. The opportunity of water injection into the anode was throughout the whole process of electroosmosis and not only the later stages (S1 Dataset).

The data points of all tests before hour 12 (half of the total ordinary electroosmosis processing time) conformed to the linear law, and the fitting formula of the amount of water discharge, *Q* [mL], and time, *t* [h], was as follows:

$$\begin{array}{ccc} Q=13.59t & \left(t<12\;h\right) & \left(R^{2}=0.98\right) \end{array}$$ (1)

If one conducts electroosmosis without the externally applied pressure, the governing equation for total volume flux of water, *q*, reads as follows [25]:

$$q=\frac{Q}{t}=k_{e}A\frac{\Delta U}{l}$$

where Δ*U* [V] is the electric potential difference, *l* [cm] is the length of soil specimen, *k*_*e*_ [cm^2^/(s•V)] is the electroosmotic permeability, A [cm^2^] is the effective cross section. In this experiment, $\frac{\Delta U}{l}=1$ V/cm, *A* = 100 cm^2^, *Q*/*t* = 13.59/3600 = 3.775 ×10^−3^ mL/s (*t* < 12 *h*). Hence, *k*_*e*_ is 3.775 × 10^−5^ cm^2^/(s•V) when time is less than 12 h and then *k*_*e*_ changes with time.

### Electric potential

**Fig 4:**
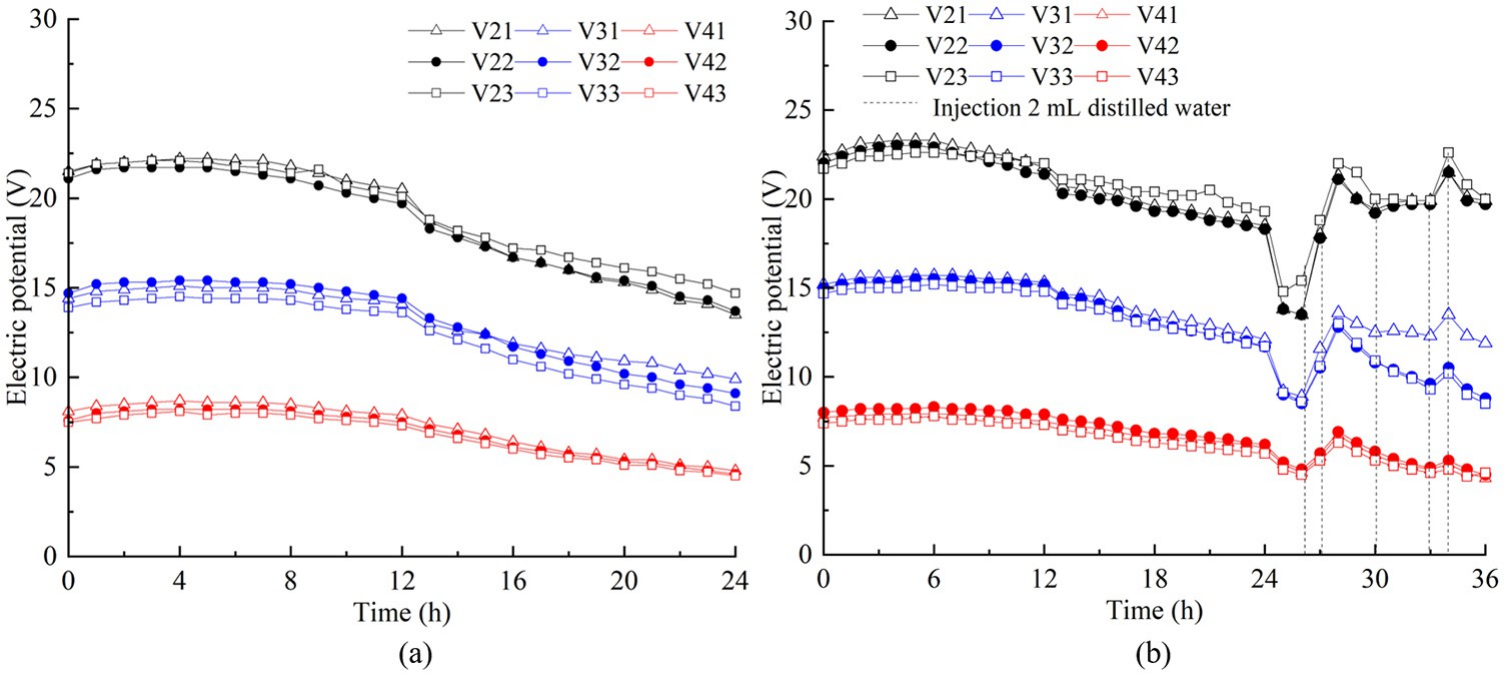
Electric potential variation. a) T6. b) T7.

The electric potential testing points were located at the soil cross sections S2, S3, and S4, as shown in Fig 2. Each cross section has three measuring points, and their depth from the soil surface is 30, 60, and 90 mm, respectively. The testing points located at S2 with the depth of 30, 60, and 90 mm were named V21, V22, and V23, respectively. The points on the S3 and S4 cross sections use the same naming convention. The electric potential change trend of T1 to T5 was similar to that of T6. Here only the monitoring results of T6 and T7 is presented, as shown in Fig 4. The change trend of T6 and T7 was similar before 24 h. After that the electric potential of T7 dropped significantly. This is because with the discharge of water in the soil, especially the soil near the anode, the interface resistance (between the electrode and the soil) and the soil resistance increase significantly, resulting in the decrease of the electric potential in the soil. A huge amount of power is lost at the later stage of ordinary electroosmosis. This is consistent with the findings from a laboratory assessment carried out by Jeyakanthan et al. [26]. However, the injection of distilled water into the anode at this stage, the situation reversed and the electric potential increased and no further decrease occurred. This demonstrated that the electrode-soil contact has been improved, which reduce the interface resistance (between the electrode and the soil) and the wastage of energy. When using the reusable EVD as electrodes, the electric potential on the same soil cross section varied little along the direction of soil depth. Also, the potential across the three cross sections decreased in almost equal proportion to the distance from the anode (S2 Dataset).

### Water content

**Fig 5:**
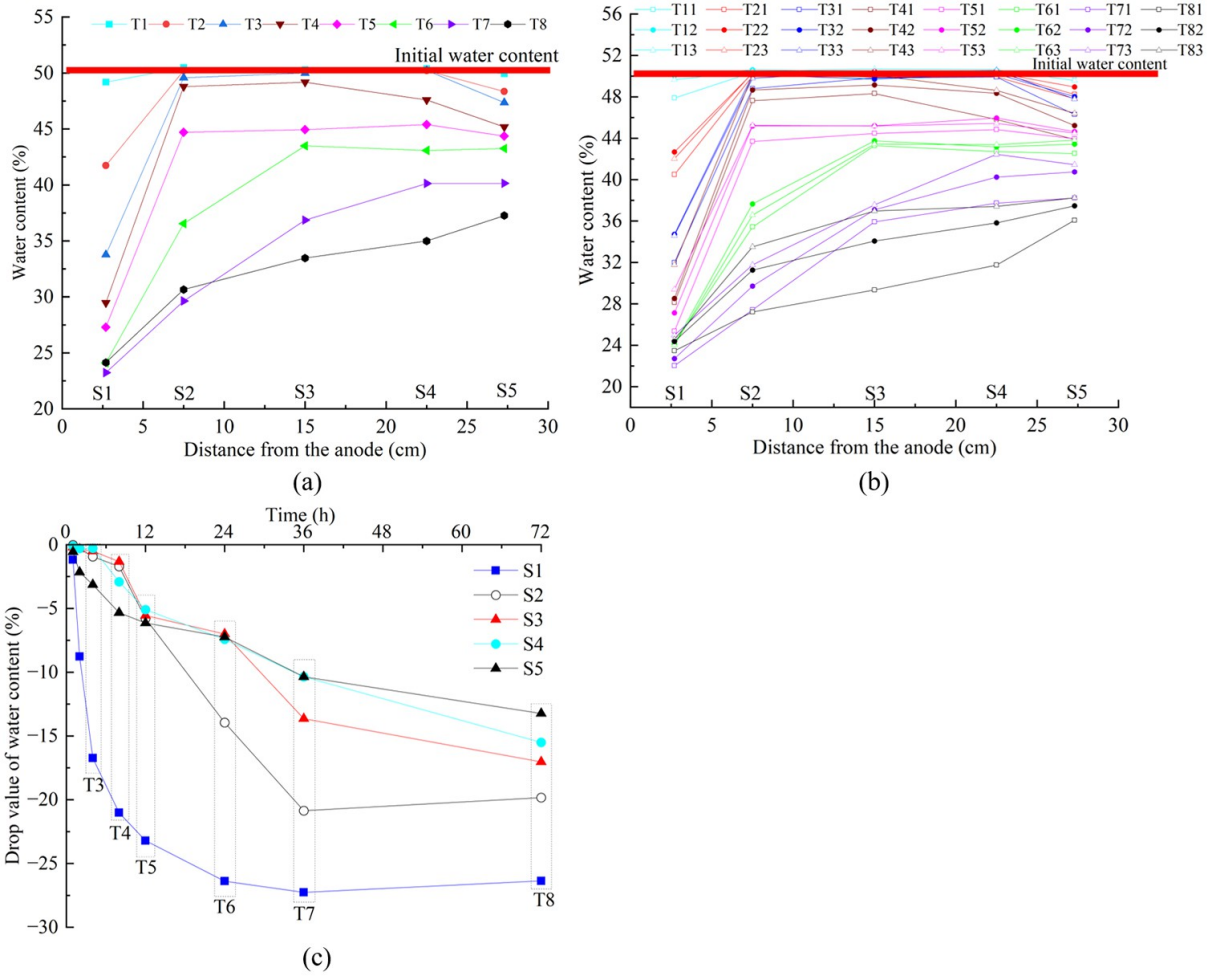
a) Average water content distribution at different soil cross sections. b) Water content distribution at different soil cross sections and depths after each test. c) Drop value of water content for each soil cross section versus time.

The water content testing points after each test were located at the soil cross sections S1 to S5, as shown in Fig 2. Each cross section has three measuring points, with depth from the soil surface of 30, 60, and 90 mm, respectively. For T1 the testing points located 30-, 60-, and 90-mm depth from the soil surface were named T11, T12, and T13, respectively. Other tests used the same naming convention. Average water content distribution at different soil cross sections for each test after a given time treatment was shown in Fig 5a. The decrease of water content for a cross section is due to the fact that the water migrating from this cross section is greater than the water migrating to it. This means water discharge toward the cathode occurred for this soil cross section (S3 Dataset).

For T1, after the soil experienced 1 h treatment, almost only the moisture content of the S1 soil cross section decreased (approximately 1.3%), which means the water discharge of T1 derived from S1. For T2-2 h and T3-4 h, the moisture content of S1 cross section decreased approximately 8.8% and 16.8%, and that of S5 soil cross section decreased 2.2% and 3.2%, respectively. The moisture content of other soil cross sections experienced no significant changed. Hence the water discharge of T2 and T3 mainly came from S1, and a minor part from S5. For T4-8 h, the moisture content of S1 and S5 soil cross section dropped further compared with T3-4h. Moreover, the moisture content of S4 soil cross section decreased to 47.6%, while that of S2 and S3 changed slightly. For T5-12 h, water discharge occurred in all cross sections toward the cathode. S1 contributed most to the total water discharge and other cross sections almost had the same amount of water drained out.

The water content distribution curve of T6-24 h is the final result after ordinary electroosmosis treatment. Compared with T5, the moisture content of each soil cross section of T6, especially S2, was further reduced. The injection of distilled water into the anode of T7 and T8 could further cause water migration in the cross section of S2-S5. Most obviously, the limiting water content of S1 is approximately 22%±2%. It is difficult to continue to further reduce after reaching this value, even when using water injection mode. The water content of S2 eventually could reduce to about 30%±2%. Compared with T7 and T8, it can be seen that injection of distilled water into the anode during the overall process of EO treatment could further induce water discharge of S3-S5.

There were some differences in moisture content at the same cross section with different depth, as shown in Fig 5b. For all tests, the moisture content with 30 mm depth was lower than that with 60 mm and 90 mm depth at the same cross sections. However, the differences of water content at the same cross section with different depth for T7 and T8, especially for T8, was significantly larger than that for T1-T6. Hence, water injection to the anode mode increased the difference of water content at the same cross section with different depth, but decreased that along the distance from the anode to the cathode.

The drop value of water content for each soil cross section versus time by synthesizing all tests was illustrated in Fig 5c. Water was transported in different soil cross sections at 12-hour intervals (the first half of the total ordinary electroosmosis processing time). The water content of S1 dropped the fastest in the first 12 h, followed by S5 and then the other three cross sections. From 12 to 24 h (the second half of the total ordinary electroosmosis processing time) the water content of S2 decreased the fastest. From 24 to 36 h the water content of S2 and S3 decreased the fastest with the same rate. The water injection mode of T7 increased the drainage rate of S3, S4, and S5, especially that of S3. The water injection mode of T8 increased the drainage rate of S4.

The drainage sequence of each cross section from the anode to the cathode versus time can be summarized as follows: At the early stage, the water discharge from the cathode mainly came from the soil near the anode (S1) and a small amount from the soil near the cathode (S5). Then the other three cross sections (S2, S3, S4) start to drainage until S2-S4 cross sections approximately reached the same water content of 45% (reduction of 5%). Thereafter, S1 and S2 continue to drain until S1 reached the limiting water content (22%±2%) and that concluded the ordinary electroosmosis treatment. The injection of water into the anode promoted S2-S5 to further drainage until S2 reached the limiting water content (30%±2%). The limiting water content varied with the soil property, electrodes materials, and voltage gradient, as can be seen from the monitoring results of water content given by Tang et al. [22] and Jayasekera [7]. But it does exist and it affects the movement of EF.

**Fig 6:**
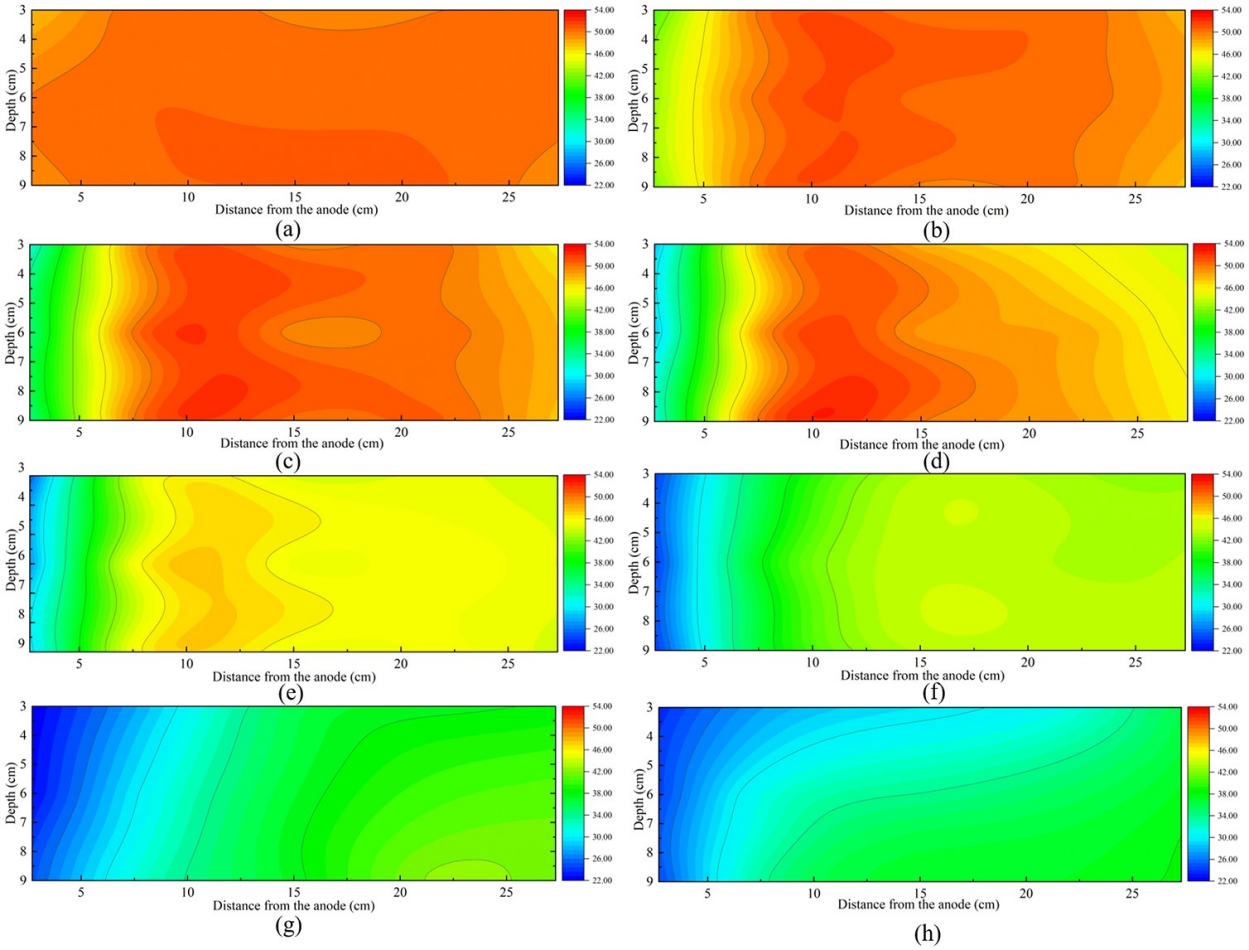
Water content contours of each test. a) T1-1 h. b) T2-2 h. c) T3-4 h. d) T4-8 h. e) T5-12 h. f) T6-24 h. g) T7-36 h. h) T8-72 h.

The convert to matrix and contour function in the Origin 8.0 software was used to map the water content contours of each test, as shown in Fig 6. These figures demonstrated the movement and accumulation of EF in the soil from the anode to the cathode under the DC power at different time. Fig 6(a) to 6(f) show the variation of water content in the soil versus time during ordinary electroosmosis treatment. The EF mainly accumulated between the distance of 7.5 cm and 15 cm from the anode before 12 h. At the end of the ordinary electroosmosis treatment the EF mainly accumulated beyond 15 cm away from the anode. The water of the soil in the middle depth moved earlier than that of the top and bottom sections. The water injection of T7 and T8 further induced the transport of EF and the water content of more areas became below 30%. The water in the upper section of the soil is transported better than the middle and the bottom sections.

Soil water content, *w* [%], during electroosmosis treatment varied with the testing points depth, *d* [cm], distance from the anode, *l* [cm], and electroosmosis treatment time, *t* [h]. Synthesizing all the data for analysis a multiple non-linear model of relations was obtained as follows:

$$w=2.02l+1.13d-1.54t-0.06l^{2}-0.07d^{2}+0.03t^{2}-0.02l\cdot d+0.02l\cdot t+0.03d\cdot t\;\left(R^{2}=0.78\right)$$ (2)

Under the specific test conditions, the moisture content of soil at different positions and times can be obtained from this equation.

### Shear strength

**Fig 7:**
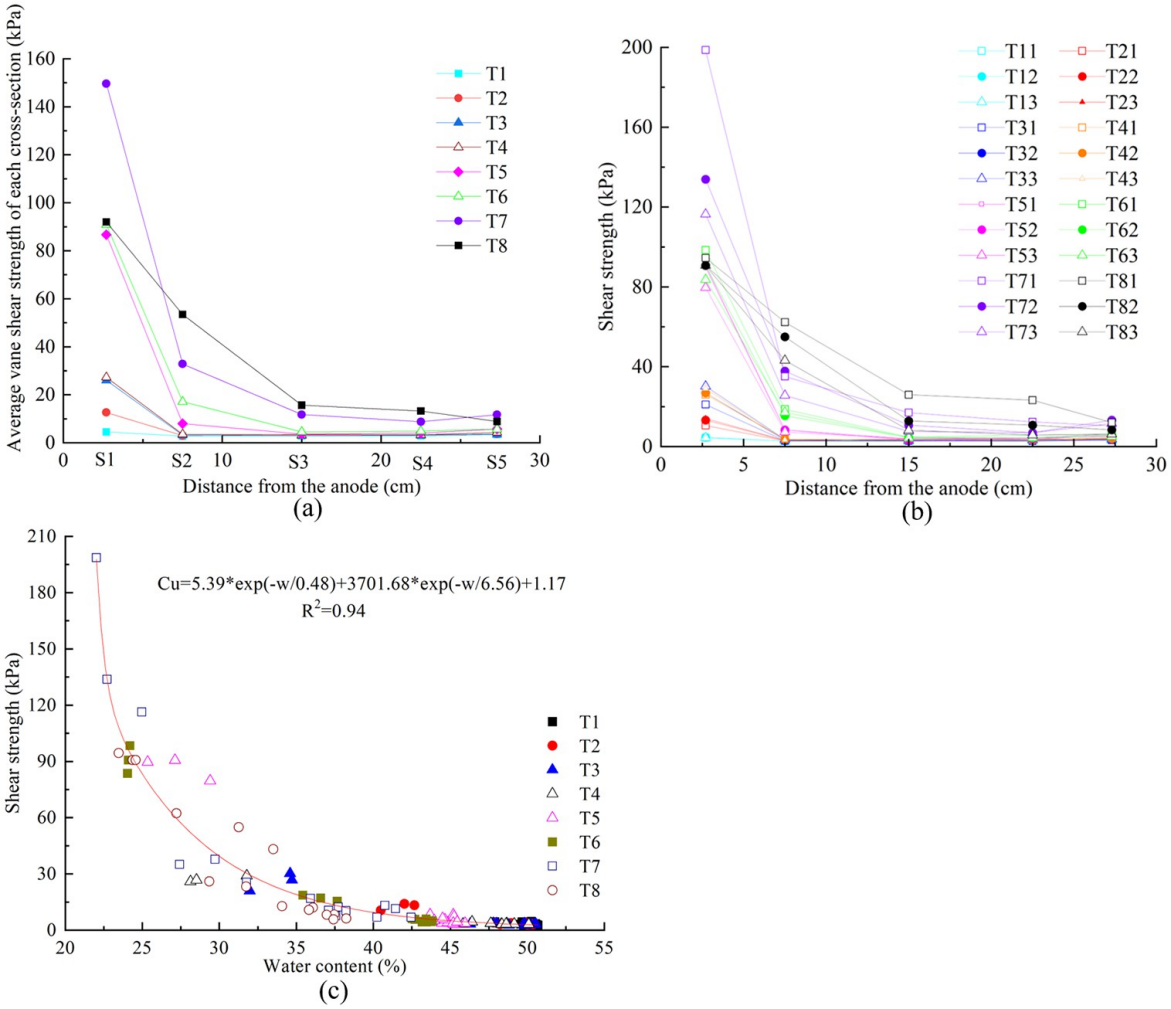
a) Average vane shear strength distribution at different soil cross sections. b) Vane shear strength distribution at different soil cross sections and depths after each test. c) Relationship between vane shear strength and water content.

The average vane shear strength distribution at different soil cross sections of each test was shown in Fig 7(a). The vane shear strength decreased from the anode to the cathode. The vane shear strength in S1 of TS7 was the largest at about 150 kPa. T5, T6, and T8 was close with almost 60 kPa, T1-T4 was lower than 30 kPa. The vane shear strength in S2 of T8 was the largest at about 53 kPa, followed by T7 at 33 kPa, T6 at 17 kPa, T5 at 8 kPa, and T1-T4 at almost 3 kPa. There was no significant difference of the vane shear strength in S3, S4, and S5 of T1-T6 and it varied between 2.9 kPa and 5.7 kPa. That of T7 and T8 was slightly higher and varied between 8.8 kPa and 15.7 kPa. Vane shear strength distribution at different soil cross sections and depths after each test was illustrated in Fig 7(b). At same cross sections the vane shear strength varied differently with depth, especially T5 and T6 at S1, T7 at S1 and S2, and T8 at S2-S4. At these cross sections the vane shear strength with 30 mm depth was 10–82 kPa, higher than that with 90 mm depth. In addition, the difference of vane shear strength in other cross sections with different depth of each test was less than 10 kPa (S4 Dataset).

According to the correspondent relationship between water content and vane shear strength at the same cross section with same depth, a scatterplot was illustrated in Fig 7(c). The data points of all tests conformed to the exponential function changing law, and the fitting formula of vane shear strength, *C*_*u*_ [kPa] and water content, *w*[%], was as follows:

$$Cu=5.39e^{\left(-\frac{w}{0.48}\right)}+3701.68e^{\left(-\frac{w}{6.56}\right)}+1.17\;\left(R^{2}=0.94\right)$$ (3)

Water content is a parameter which was easy to be obtained in laboratory soil tests. According to the above formula, the shear strength of EO treated soil can be deduced.

### Atterberg limits

**Fig 8:**
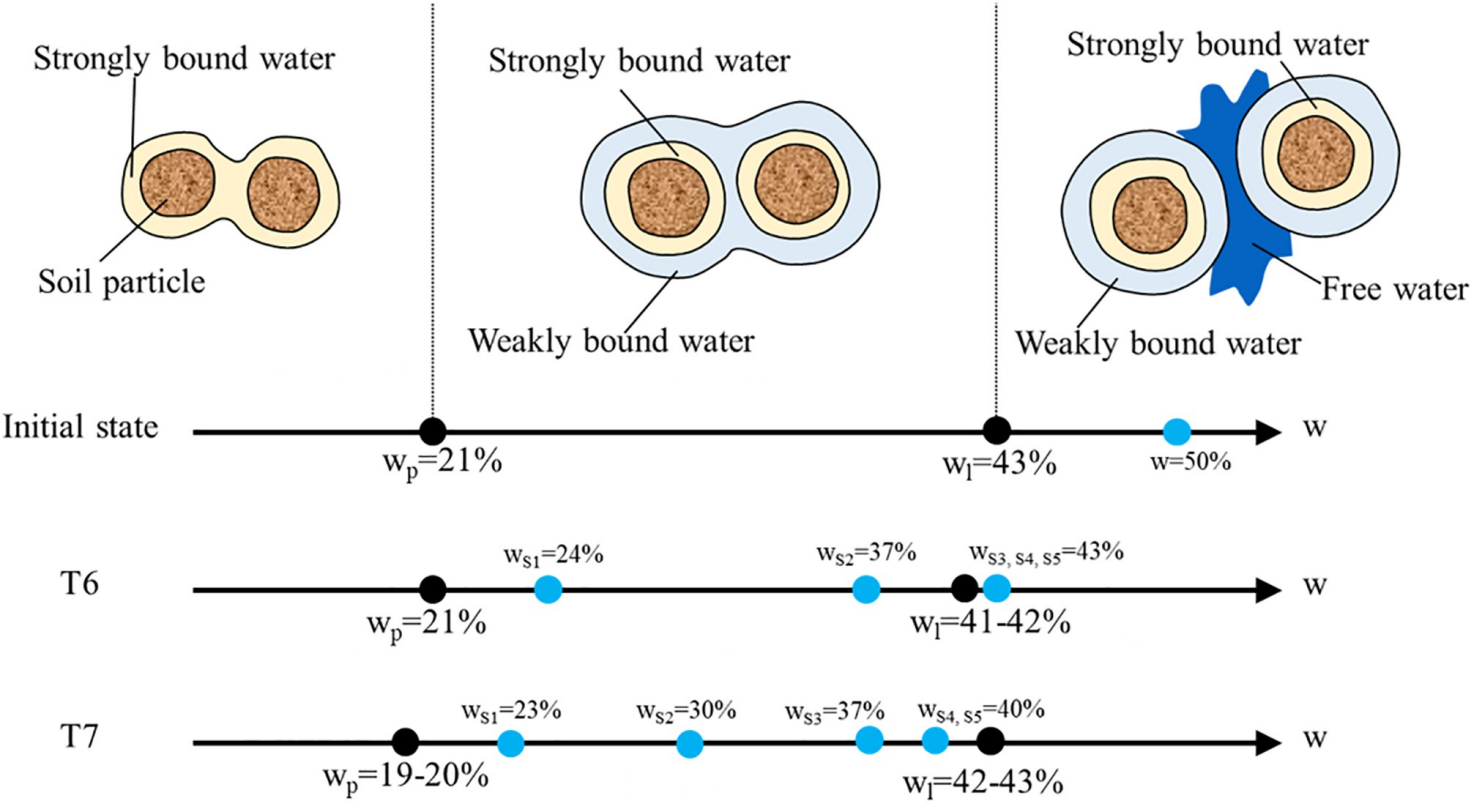
Atterberg limit and water content of T6 and T7.

The Atterberg limits reflect the form of the water in the soil, as shown in Fig 8. If the soil water content is lower than the plastic limit, the water exists as strongly bound water surrounding the soil particles in a solid or semi-solid state. The strongly bound water film is the largest at the plastic limit. When the soil water content between the plastic limit and the liquid limit, weakly bound water and strongly bound water exists simultaneously in a pluripotent state. If the water content was larger than the liquid limit, free water would appear in the soil and the soil would be in a flow state [27]. Before treatment the soil plastic limit and liquid limit was 21% and 43%, respectively. The soil initial water content was about 50% in a flow state. After electroosmosis treatment the Atterberg limits of the soil near the anode, cathode, and the middle location between the electrodes were tested. The overall change of Atterberg limits of T6 and T7 was shown in Fig 8. It appeared that there was a slight decrease of T6 and T7, this may be related to the testing error or the change of soil chemical composition [28].

For T6, the average water content of S1 and S2 was between the plastic limit and the liquid limit, that of S3, S4, and S5 was slightly larger than the liquid limit. This indicated that after ordinary electroosmosis treatment only bound water existed in half of the soil near the anode and a small amount of free water existed in the other half of soil. For T7, the average water content of all cross sections was between the plastic limit and the liquid limit. This indicated that after electroosmosis with water injection treatment only bound water existed in the soil. Moreover, the moisture content of the soil cannot be reduced below the plastic limit.

### Coefficient of variation of electroosmosis treated soil

**Fig 9:**
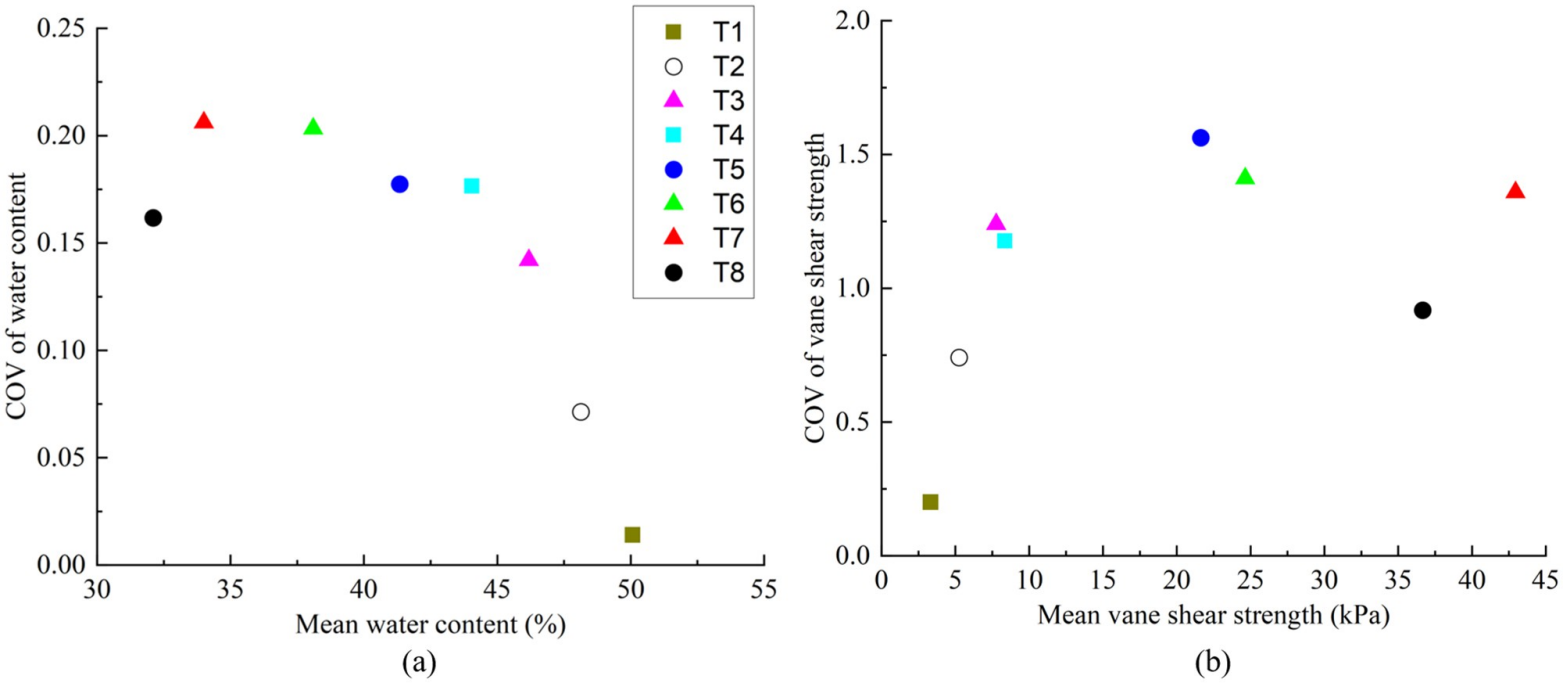
a) COV of water content versus mean water content. b) COV of vane shear strength versus mean vane shear strength.

Phoon and Kulhawy [29] indicated that a designer should select the appropriate resistance factors based on the variability of the soil at a specific site. Coefficient of variation (COV) is a typical parameter to estimate soil property variability, which can be obtained by normalizing the standard deviation with respect to the mean soil property. Fig 9 illustrated the variation of the COV for the water content (*w*) and vane shear strength (*C*_*u*_) versus the mean *w* and *C*_*u*_ for each test. It can be seen that in the process of ordinary electroosmosis treatment the COV of *w* increased with the decrease of soil mean *w*, and the COV of *C*_*u*_ increased with the increase of soil mean *C*_*u*_. The water injection mode also influences the final COV and mean for *w* and *C*_*u*_. In contrast, water injection into the anode during the whole process of electroosmosis gives better COV and mean for *w* and *C*_*u*_.

## Conclusions

It is urgent to improve the reinforcement quality and efficiency of electroosmosis and to reduce energy consumption and cost through technological innovation and mechanism research. This is of great significance to promote its engineering application. In this investigation, an experimental program was conducted to evaluate the behaviour or effect of EF on soft clay using EKG as electrodes. From the results, the following conclusions can be drawn.

(1) Before half of the total ordinary electroosmosis processing time, the amount of water discharge conformed to the linear law. Water injection into the anode during the whole process of electroosmosis can evidently alleviated EF accumulation and greatly improved the water discharge of soft clay. Moreover, the injection of distilled water into the anode helped sustain the electric potential, which demonstrated that the electrode-soil contact has been improved to reduce the wastage of energy.
(2) The drainage sequence of each cross section from the anode to the cathode versus time can be summarized as follows: At the early stage, the water discharge from the cathode mainly came from the soil near the anode (S1) and a small amount from the soil near the cathode (S5). Then other three cross sections (S2, S3, S4) start to drainage until S2-S4 cross sections approximately reached the same water content 45% (reduction of 5%). Thereafter, S1 and S2 continue to drain until S1 reached the limiting water content (22%±2%) and that concluded the ordinary electroosmosis treatment. The injection of water into the anode promoted S2-S5 to further drainage until S2 reached the limiting water content (30%±2%).
(3) A multiple non-linear model about relations between the soil water content (*w*) with the testing points depth (*d*), distance from the anode (*l*), and electroosmosis treatment time (*t*) was obtained. The vane shear strength (*C*_*u*_) and its corresponding water content (*w*) of all tests conformed to the exponential function changing law and a fitting formula was given. After electroosmosis with water injection treatment only bound water existed in the soil, but the moisture content cannot be reduced below the plastic limit. Water injection into the anode during the whole process of electroosmosis gives better COV and mean for *w* and *C*_*u*_.

In general, electroosmosis with water injection into the anode technique is an effective method for the dewatering and consolidating of soft clay. The application of electroosmosis technology has been relatively mature. On this basis, additional water pipes need to be laid to inject water into the anodes. The end of the pipe leads to the anode and is tied together with the anode to fix it, and the other end is connected to the water pump to deliver the water to each anode. In addition, the timing and amount of water injection should be determined according to the drainage rate at the cathode. When the discharge rate of the cathode decreases obviously, water can be injected at the anode. The amount of water injected should be adjusted from less to more to avoid exceeding the drainage amount.

## Supporting information

S1 Dataset Water discharge. (XLSX)

S2 Dataset Electric potential. (XLSX)

S3 Dataset Water content. (XLSX)

S4 Dataset Shear strength. (XLSX)
